# Mechanisms of Drug Resistance in Relapse and Refractory Multiple Myeloma

**DOI:** 10.1155/2015/341430

**Published:** 2015-11-16

**Authors:** Wen-Chi Yang, Sheng-Fung Lin

**Affiliations:** ^1^Division of Hematology and Medical Oncology, Department of Internal Medicine, Yuan's General Hospital, No. 162, Cheng Kung 1st Road, Kaohsiung 802, Taiwan; ^2^Molecular Medicine Lab, Yuan's General Hospital, No. 162, Cheng Kung 1st Road, Kaohsiung 802, Taiwan; ^3^Faculty of Meiho University, No. 23, Pingguang Road, Neipu, Pingtung 912, Taiwan; ^4^Faculty of Medicine, College of Medicine, Kaohsiung Medical University, No. 100, Tzyou 1st Road, Kaohsiung 807, Taiwan; ^5^Division of Hematology and Oncology, Department of Internal Medicine, Kaohsiung Medical University Hospital, No. 100, Tzyou 1st Road, Kaohsiung 807, Taiwan

## Abstract

Multiple myeloma (MM) is a hematological malignancy that remains incurable because most patients eventually relapse or become refractory to current treatments. Although the treatments have improved, the major problem in MM is resistance to therapy. Clonal evolution of MM cells and bone marrow microenvironment changes contribute to drug resistance. Some mechanisms affect both MM cells and microenvironment, including the up- and downregulation of microRNAs and programmed death factor 1 (PD-1)/PD-L1 interaction. Here, we review the pathogenesis of MM cells and bone marrow microenvironment and highlight possible drug resistance mechanisms. We also review a potential molecular targeting treatment and immunotherapy for patients with refractory or relapse MM.

## 1. Introduction

Multiple myeloma (MM) is a clonal B-cell malignancy that is characterized by the proliferation of a plasma cell clone that produces a monoclonal immunoglobulin. MM leads to end-organ damage diseases such as anemia, hypocalcemia, renal insufficiency, or osteolytic bone lesions [1]. The incidence of MM is around 15,000 per year in the US and Europe, and the median survival is about 4-5 years [2]. In addition to the International and Durie-Salmon staging systems [1], biological markers, including cytogenetic abnormalities such as presence of hypodiploidy, t(4;14), t(14;16), del(17p), and del(13), serum *β*2-microglobulin levels greater than 2.5 mg/L, an elevated plasma cell labeling index, and detection of circulating plasma cells, are predictors of poor prognosis in newly diagnosed MM patients [1, 3–10]. Over the past decade, new therapeutic strategies for MM have been developed on the basis of a deeper understanding of the biology of myeloma cells and their interaction with the bone marrow (BM) microenvironment. These therapies include novel proteasome inhibitor agents such as bortezomib [11, 12] and immunomodulatory drugs such as thalidomide [13, 14] and lenalidomide [15, 16]. Implementation of these therapies has led to increased longevity in MM patients, with median survival of over 5 years [17]. However, many patients still relapse or become refractory to treatment [18]; therefore, MM still is an incurable disease, and understanding the disease mechanism is important, specifically for the development of effective treatments.

## 2. Myeloma Tumor Cells and the BM Microenvironment

Plasma cells are derived from hematopoietic cells via Ig VDJ rearrangement, somatic mutation, and Ig class switching [19]. Myeloma cells are postgerminal, long-lived plasma cells with mutated homogeneous clonal sequences [19, 20]. MM cells express CD38 and CD138 antigens on the cell surface but lack CD45 and surface Ig expression [19]. Chromosomal alterations have been detected by conventional karyotyping, interphase fluorescence in situ hybridization (FISH) [21, 22], and spectral karyotyping analysis [23] in 30%–50% of MM patients. The results of these analyses have suggested two different pathways of pathogenesis: (1) nonhyperdiploid tumors with a very high incidence of IgH translocations involving five well-defined recurrent chromosomal translocation areas (11q13 [cyclin D1], 6p21 [cyclin D3], 4p16 [fibroblast growth factor receptor 3, FGFR3], multiple myeloma SET domain [MMSET], 16q23 [c-maf], and 20q11 [mafB]) [24] and relatively high incidence of chromosome 13/13q14 loss and (2) hyperdiploid tumors associated with multiple trisomies involving chromosomes 3, 5, 7, 9, 11, 15, 19, and 21, but low incidence of both chromosome 13/13q14 loss and IgH translocation [25]. These chromosomal alterations lead to dysregulation of cyclin D and selective expansion during interaction with BM stromal cells (BMSCs), which produce interleukin-6 (IL-6) and other cytokines [25].

The BM microenvironment is important for MM pathogenesis. The very-late antigen-4 (VLA-4) on MM cells binds to fibronectin in the serum, and the lymphocyte function associated antigen-1 (LFA-1) on MM cells binds to intercellular adhesion molecule-1 (ICAM1) on BMSCs [26], causing MM cells to home in to the BM. Other cytokines such as tumor-necrosis factor-*α* (TNF-*α*) in the BM can modulate the adhesion of MM cells in the BM by inducing nuclear factor- (NF-) *κ*B. NF-*κ*B-dependent upregulation of cell surface adhesion molecules such as ICAM1 and vascular cell-adhesion molecule-1 (VCAM1), on both MM cells and BMSCs, increases the binding capacity of tumor cells and BMSCs and induces the transcription and secretion of cytokines such as IL-6 and VEGF in BMSCs [27]. Cytokines in the BM microenvironment, such as IL-6, insulin-like growth factor-1 (IGF-1), vascular endothelial growth factor (VEGF), and TNF-*α*, mediate the growth of MM cells. However, IL-6, IGF-1, and IL-21 are associated with tumor cell survival and resistance to apoptosis [28–36]. This association is mediated through the Janus kinase (JAK)/signal transducer and activator of transcription 3 (STAT3) and phosphatidylinositol 3-kinase (PI3K)/AKT pathways. The proliferation of MM cells is triggered by cytokines such as IL-6, IGF-1, VEGF, TNF-*α*, stromal cell derived factor-1*α* (SDF-1*α*), and IL-21 and is mediated through the RAF/mitogen-activated protein kinase kinase (MEK)/p42/p44/mitogen-activated protein kinase (MAPK) signaling cascade [27, 30, 32, 36–39]. VEGF and SDF-1*α* play important roles in cell migration, and the migration of MM cells is mediated through a protein kinase C- (PKC-) dependent, p42/p44/MAPK-dependent pathway [37, 40, 41].

Immune compromise is a major complication in MM patients. Programmed death receptor-1 (PD-1, CD279) is a receptor of the Ig superfamily that negatively regulates T cell antigen receptor signaling by interacting with specific ligands (PD-L1). PD-1 is suggested to play a role in the maintenance of self-tolerance. PD-1 is induced on activated T cells and is expressed on exhausted T cells [42]. Engagement of PD-1 by its ligands, PD-L1 (B7-H1, CD274) or PD-L2 (B7DC, CD273), results in the activation of phosphatases that deactivate signals emanating from the T-cell receptor [43]. Moreover, PD-1 engagement upregulates the expression of basic leucine ATF-like transcription factor (BATF), which in turn impairs T-cell proliferation and cytokine secretion [44]. PD-L1 plays a crucial role in the evasion of the host immune system by tumor cells [45]. PD-L1 is more ubiquitous than PD-L2, and MM cells express elevated levels of PD-L1 [46]. T cells from myeloma-bearing mice and MM patients express higher levels of PD-1. These PD-1-positive T cells were found to be exhausted and produced IL-10 [47, 48]. Stimulation by interferon-*γ* (IFN-*γ*) and Toll-like receptor (TLR) ligands upregulated PD-L1 expression in MM cells from MM patients via the MyD88/TRAF6, MEK, and STAT1 pathway [46].

MicroRNAs (miRNAs) play crucial roles in cancer progression [49], and many miRNAs are deregulated in multiple myeloma. Al Masri et al. reported that the expression levels of* miR-125b*,* miR-133a*,* miR-1*, and* miR-124a* vary in multiple myeloma [50]. Among the 464 miRNAs analyzed, 95 were shown to be expressed at higher levels in patients with MM than in healthy donors [51]; this dysregulation of miRNA expression included upregulation of* miR-let-7a*,* miR-16*,* miR-17-5p*,* miR-19b*,* miR-21*,* miR-531*,* miR-335*,* miR-342-3p*,* miR-25*,* miR-32*,* miR-20a*, and* miR-93*; increased expression of the miRNA cluster containing* miR-106a*,* miR-106b*,* miR-181a*,* miR-19b*,* miR-181b*,* miR-92a*, and* miR-17-92* [52–54]; and downregulation of* miR-372*,* miR-143*, and* miR-155* [52]. In patients with monoclonal gammopathy of undetermined significance (MGUS), 41 miRNAs were shown to be upregulated, with* miR-181*,* miR-21*,* miR-106a*,* miR-25*, and* miR-93* showing the greatest upregulation, whereas seven miRNAs were shown to be downregulated, compared with the levels in healthy plasma cells [55]. These abnormally regulated miRNAs target genes regulating the cell cycle, apoptosis, survival, and cell growth; for example, the* miR-17-92* cluster regulates Bcl-2 [56],* miR-29b* regulates MCL1 [57],* miR-21* regulates STAT3 in an IL-6-dependent manner [53], and* miR-125b* regulates BLIMP1 and IRF4 [58].

## 3. Current Biological Based Therapies for MM

Improved understanding of the pathogenesis and importance of the BM microenvironment in MM has led to the development of two therapeutic categories for MM treatment: proteasome inhibitors and immunomodulatory drugs. These therapies have significantly improved treatment response and survival in MM patients.

### 3.1. Proteasome Inhibitor

Bortezomib is a proteasome inhibitor that inhibits the activity of the 26S proteasome [59]. Bortezomib blocks the degradation of I*κ*B*α*, an inhibitory protein that is constitutively bound to cytosolic NF-*κ*B, thereby inhibiting the nuclear translocation and activation of NF-*κ*B. Bortezomib induces apoptosis by activating caspase-8 and caspase-9 in drug-resistant MM cell lines and primary cancer cells derived from MM patients. Moreover, bortezomib downregulates the expression of adhesion molecules on MM cells and BMSCs and their related binding. Bortezomib also inhibits IL-6 and/or BMSC/MM cell adherence-induced p42/p44 MAPK phosphorylation and proliferation of MM cells [60, 61].

Bortezomib has received full FDA approval for the treatment of relapse, refractory, and newly diagnosed MM patients based on the results of phase III trials [11, 12]. Treatment regimens including bortezomib have become the standard treatment for multiple myeloma patients, particularly for hematopoietic stem cell transplantation-eligible patients, because of the improved response rate and survival compared to chemotherapy and steroid treatment alone [11, 62–75].

### 3.2. Thalidomide and IMiDs

Thalidomide and the more potent second-generation thalidomide analogues, IMiDs, target myeloma cells in the BM microenvironment. They inhibit TNF-*α* production [75, 76] and angiogenesis by blocking the angiogenic growth factors, basic fibroblast growth factor (bFGF), and VEGF [77]. Specifically, these agents trigger caspase-8-mediated apoptosis and enhance both caspase-8-mediated MM cell apoptosis, triggered by FAS or TRAIL, and caspase-9-mediated MM cell killing, triggered by dexamethasone [78–80]. They also block the induction of cytokines such as IGF-1 and IL-6 and VEGF secretion triggered by MM cell adherence to BMSCs. In addition, they inhibit angiogenesis and augment natural killer cell activity against autologous MM cells [79–82]. Several clinical trials have demonstrated the benefits of using regimens involving thalidomide or IMiDs (lenalidomide) for MM treatment, particularly in combination with proteasome inhibitors [15, 16, 63–66, 69–71, 74, 83–96]. This combined therapy has become the standard regimen for MM treatment. Pomalidomide therapy has afforded prolonged progression-free survival in patients who relapsed or became refractory to lenalidomide treatment [97].

The choice of therapy for patients is influenced by a variety of factors, including age, comorbidities, and eligibility for stem cell transplantation. Treatment strategies for MM patients include two-drug regimens such as bortezomib-dexamethasone [62], lenalidomide-dexamethasone [15, 16, 86, 94], or thalidomide-dexamethasone [63, 84, 92, 93] and three-drug regimens such as bortezomib-thalidomide-dexamethasone [63–66], bortezomib-melphalan-prednisone [68, 69], or lenalidomide-bortezomib-dexamethasone (RVD) [98]. However, RVD has shown the most promising effect.

## 4. Mechanisms of Drug Resistance

**Figure 1:**
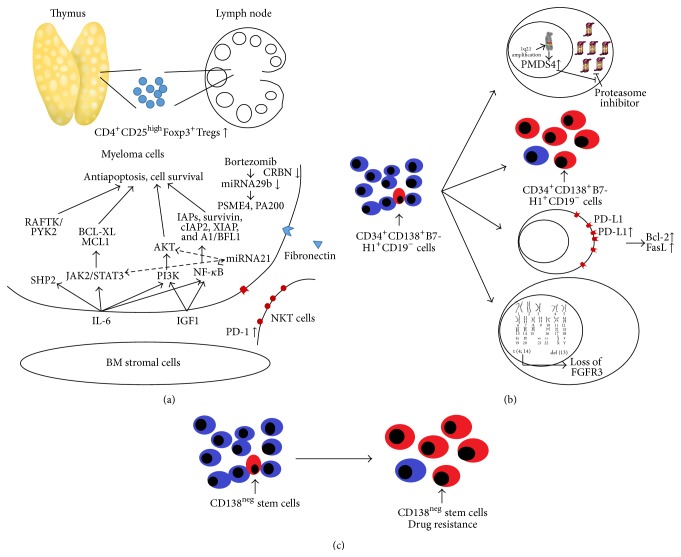
The mechanism of drug resistance of refractory and relapse multiple myeloma. (a) Microenvironment, (b) clonal evolution of myeloma cells, and (c) cancer stem cell.

During conventional chemotherapy such as treatment with vincristine and doxorubicin, accumulation of drugs induces the expression of multidrug resistance (MDR) genes and p-glycoprotein in tumor cells [99–101]. The BM microenvironment can confer drug resistance through two major mechanisms (Figure 1(a)) [102]: (1) tumor cell adhesion, which involves MM cell binding to fibronectin, which in turn induces KIP1 and G1 growth arrest and confers cell-adhesion mediated drug resistance [103, 104] and (2) cytokine-mediated antiapoptotic sequelae, which involve the induction of JAK/STAT and PI3K/AKT signaling by cytokines in the BM microenvironment, which in turn mediates resistance to conventional and novel therapies. IL-6 induces resistance to dexamethasone by activating JAK/STAT signaling and upregulating the antiapoptotic proteins, BCL-XL [105, 106] and myeloid cell leukemia sequence-1 (MCL1) [107, 108]. IL-6 also activates SRC-homology tyrosine phosphatase 2 (SHP2), which blocks dexamethasone-induced activation of RAFTK and apoptosis [109]. Both IL-6 and IGF-1 inhibit drug-induced apoptosis of MM cells through PI3K/AKT signaling and NF-*κ*B activation, which in turn induces the intracellular expression of downstream inhibitor of apoptosis proteins (IAPs), FLICE-inhibitory protein (FLIP), survival, cellular inhibitor of apoptosis-2 (cIAP2), A1/BFL1, and X-linked inhibitor of apoptosis protein (XIAP) [32, 35, 110, 111]. Neither bortezomib nor thalidomide/IMiDs can block JAK/STAT or PI3K/AKT signaling [102].

MicroRNAs play a key role in multidrug resistance in cancers by modulating drug transporter-related proteins, cell cycle-related proteins, drug targets, autophagy, the tumor microenvironment, cell survival signaling, and apoptosis pathways [112, 113]. Roccaro et al. reported that the expression of* miR-15a* and* miR-16* decreased, while the expression of* miR-221*,* miR-222*,* miR-382*,* miR-181a*, and* miR-181b* increased in patients with relapse/refractory MM compared with the levels in healthy volunteers [114]. Moreover,* miR-15a* and* miR-16* regulate the cell cycle by inhibiting the expression of cyclin D1, cyclin D2, and CDC25A and the phosphorylation of Rb, resulting in G_1_ arrest.* miR-15a* and* miR-16* can also reduce the expression of Bcl-2. Cells transfected with pre-*miRNA-15a* and pre-*miRNA-16-1* exhibit the following effects: (1) increased phosphorylation of the inhibitory protein I*κ*B in the cytoplasm, indicating involvement of these miRNAs in both the canonical and noncanonical NF-*κ*B pathways; (2) significantly decreased VEGF secretion, suggesting an antiangiogenic role for these miRNAs; (3) and inhibition of migration in response to SDF-1. Significant inhibition of the adhesion of MM to primary BM stromal cells upon application of these miRNAs has been confirmed in mouse models. In addition, the* miR-15a*/*miR-16-1* cluster upregulates several genes, including* NEDD9*,* Snai2*,* MALAT1*, and* VEGF*, and leads to the inhibition of tumor progression by enhancing tumor cell survival, metastasis, and the angiogenic properties of MM cells [115]. Neri et al. reported the dysregulation of several miRNAs related to bortezomib resistance, including the overexpression of* miR-155*,* miR-342-3p*,* miR-181a*,* miR-181b*,* miR-128*, and* miR-20b* and the downregulation of* miR-let-7b*,* miR-let-7i*,* miR-let-7d*,* miR-let-7c*,* miR-222*,* miR-221*,* miR-23a*,* miR-27a*, and* miR-29a* [116]. The predicted targets genes include genes involved in cell cycle regulation, cell growth, apoptosis, and the ubiquitin-conjugation pathways [115].* miR-21* targets Rho-B, PTEN, and BTG2 and controls STAT-3/IL-6-dependent pathways as well as AKT and NF-*κ*B signaling via myeloma cell adhesion to BMSCs [117–119].* miR-21* inhibitor exhibits synergistic effects with dexamethasone, doxorubicin, and bortezomib [117], indicating that* miR-21* may be involved in mediating drug resistance. Another miRNA,* miR-29b*, has been shown to target* PSME4*, which encodes the proteasome activator PA200; this miRNA is significantly downregulated in bortezomib-resistant cells and in cells resistant to second-generation proteasome inhibitors, for example, carfilzomib and ixazomib [120]. Bortezomib promotes the accumulation of polyubiquitinated proteins and induces aggresome and autophagosome formation to promote protein clearance, tumor survival, and relative drug resistance. Activating transcription factor 4 (ATF4), an endoplasmic reticulum-resident transmembrane protein, and microtubule-associated protein 1 light chain 3B (LC3B), one of the key factors in autophagosome formation, play a critical role in activating autophagy and protecting breast cancer cells from bortezomib-induced cell death, representing another potential mechanism of resistance to bortezomib [121].

Clonal evolution of MM cells is another possible mechanism of drug resistance (Figure 1(b)). Hyperexpression of the proteasome-related gene,* PSMD4*, is highly sensitive to chromosome 1q21 amplification and is reported to be associated with bortezomib resistance [122]. MM with gain of chromosome 1q has demonstrated poor prognosis [123], and patients with relapse or refractory MM who received treatment with lenalidomide and dexamethasone in the presence of del(13) and t(4;14) chromosomal abnormalities exhibited lower response rates and shorter median progression-free survival (PFS) [4]. Chromosome t(4;14) is likely to evolve over time, first to a chimeric and ultimately to an unbalanced translocation, with the associated loss of FGFR3 expression, which indicates disease progression [124]. B7-H1 (PD-L1) expression is upregulated on the surface of cells from MM patients. Compared to B7-H1^−^ human myeloma cell lines (HMCLs), B7-H1^+^ HMCLs were found to be more proliferative and less susceptible to dexamethasone and melphalan treatment and were accompanied by higher Bcl-2 and FasL expression [125, 126]. The expression levels of PD-L1 were found to be upregulated after myeloma patients relapsed or became refractory to therapy [126]. Kuranda et al. also reported that a small subpopulation of cycling CD34^+^CD138^+^B7-H1^+^CD19^−^ plasma cells were found in MM patients, and these cells often expressed Ki67, a marker for proliferation, and limited the clinical benefits of autologous CD34^+^ cell transplantation [127]. However, a population of suppressive CD4^+^CD25^high^Foxp3^+^ regulatory T cells (Tregs) accumulated in the thymus and lymphoid peripheral organs during disease progression [128].

Another mechanism of drug resistance involves epigenetic inactivation of genes such as* RASD1*. Methylation of* RASD1*, which encodes a Ras family protein that is induced by dexamethasone and suppresses cell growth, was found to be associated with its inactivation, which correlated with resistance to dexamethasone [129].

The concept of cancer stem cells was introduced in the late 1990s. Traditionally, cancer cells that survive chemotherapy and acquire drug resistance are thought to give rise to a population of drug-resistant cancer cells through modulation of mechanisms such as drug inactivation, changes in the expression of cellular targets, suppression of drug accumulation, and inhibition of drug activation [130–132]. The Notch, Wnt, and Hedgehog pathways play a role in regulating normal stem cells and the pathogenesis of a wide variety of human cancers, including MM [133–137]. Aberrant activation of Hedgehog signaling has been identified in MM. Pathway activation by ligands results in the expansion of immature myeloma cells, whereas the inhibition of signaling with a ligand-neutralizing monoclonal antibody or antagonists of the positive mediator of the pathway signaling induces plasma cell differentiation [137, 138]. Matsui et al. identified a group of CD138^neg^ MM cells that possess high drug efflux capacity and intracellular drug detoxification activity. Alternatively, MM cells expressing the memory B-cell markers CD20 and CD27 from the peripheral blood could give rise to clonogenic MM growth* in vitro* and in SCID/NOD mice [139]. These data support the hypothesis that MM cells exhibit stem cell characteristics.

Cereblon (CRBN) is the primary target of thalidomide teratogenicity [140]. Thalidomide binds to CRBN, alters the function of the E3 ubiquitin ligase complex, and induces downstream effects, including cell cycle arrest caused by the upregulation of the cyclin-dependent kinase inhibitor p21^WAF-15^ and the downregulation of interferon regulatory factor 4 (IRF4), which targets critical genes, including* MYC*,* CDK6*, and* CASP* [141–143]. CRBN is also required for the anti-MM action of the thalidomide derivatives lenalidomide and pomalidomide; decreasing the expression of* CRBN* results in resistance to IMiDs, as evidenced by both* in vitro* and clinical studies [144–148]. However, the majority of patients with low CRBN levels do not harbor genomic mutations [149].

## 5. Potential New Therapies for Refractory and Relapse MM Patients

### 5.1. Second-Generation Inhibitors of the Ubiquitin-Proteasome Cascade [150]

Recently, potent inhibitors with chymotryptic activity have been developed. These include carfilzomib, ONX 0912, and MLN 9708 [151, 152], which can overcome bortezomib resistance, as demonstrated in preclinical and early clinical trials. Carfilzomib was approved by the FDA in July 2012 to treat relapse and refractory MM patients who had received prior treatment with bortezomib and thalidomide/lenalidomide [153, 154]. The safety and efficacy of carfilzomib were demonstrated in the PX-171-003-A1 trial, a prospective phase II trial in patients with relapse or refractory MM who had received at least two prior therapies, including a proteasome inhibitor and an immunomodulatory agent [155]. A randomized phase III clinical trial comparing carfilzomib-lenalidomide-dexamethasone and lenalidomide-dexamethasone treatment regimens in patients with relapse MM [156] and another randomized phase III clinical trial comparing carfilzomib-dexamethasone and bortezomib-dexamethasone in patients with relapse MM [157] are ongoing. ONX 0912 [151] and MLN 9708 [158] are novel orally bioavailable proteasome inhibitors that trigger apoptosis by activating caspase-3, caspase-8, and caspase-9. Ongoing phase I and II clinical trials for these inhibitors have shown promising results [159–161].

P5091 is another second-generation proteasome inhibitor that targets the deubiquitinating enzyme USP7 and induces apoptosis in MM cells resistant to conventional and bortezomib therapies [162]. NPI-0052 is a broader proteasome inhibitor that targets chymotryptic, tryptic, and caspase-like activities to overcome bortezomib resistance in preclinical studies [163]. PR-924, an inhibitor of the LMP-7 immunoproteasome subunit, also blocks MM cell growth* in vitro* and* in vivo* [164].

### 5.2. Immunomodulatory Agents [150]

Pomalidomide is a distinct oral IMiD immunomodulatory agent with direct antimyeloma, stromal-support inhibitory, and immunomodulatory effects. Pomalidomide can synergize* in vitro* with proteasome inhibitors such as bortezomib [79]. Phase 1 clinical studies of pomalidomide in combination with low-dose dexamethasone have demonstrated the effectiveness of this therapy in MM patients who were resistant to other agents, including thalidomide, lenalidomide, and bortezomib [165, 166]. The pivotal multicenter, open-label, randomized phase III trial, MM-003, compared pomalidomide and low-dose dexamethasone with high-dose dexamethasone in 455 patients with refractory or relapse MM after failure of bortezomib and lenalidomide treatment. Pomalidomide and low-dose dexamethasone induced better progression-free survival and favorable overall survival without cross-resistance of prior treatment of lenalidomide and/or thalidomide [167].

### 5.3. PD-1/PD-L1 in Multiple Myeloma [150]

PD-L1 expression is increased in MM cells, and PD-1 is expressed on a relatively large number of T cells in myeloma-bearing mice, but only in sites of tumor accumulation [48]. Binding of PD-L1 to PD-1 expressed on the surface of activated T cells delivers an inhibitory signal, thereby reducing cytokine production and proliferation [168]. Preclinical data have confirmed the important role of the PD-1 pathway in immune evasion by MM cells [46, 48, 168]. In phase I clinical trials, objective responses were observed in patients with melanoma, renal cell carcinoma, and non-small cell lung cancer, who underwent immunotherapy with an anti-PD-1 monoclonal antibody [169–172]. In addition, an anti-PD-L1 monoclonal antibody exhibited antitumor activity in patients with melanoma, renal cell carcinoma, non-small cell lung cancer, and ovarian cancer [172, 173]. Pidilizumab (CT-011), an anti-PD1 antibody, enhances NK-cell activity against autologous, primary MM cells. In addition, lenalidomide downregulates PD-L1 in MM cells and may augment CT-011-mediated enhancement of NK-cell activity against MM [47]. However, another anti-PD1 antibody, nivolumab (BMS-936558), did not show objective responses in MM [174]. This may be attributed to the fact that the mechanism of action of T-cell activity against MM cells does not involve PD-1/PD-L1 interaction. Clonal cytotoxic CD8^+^ T cells are the only definitive T cells that have a protective role and impact on survival in MM [175]. Cytotoxic T-cell clones (CD57^+^CD28^−^TCRV*β* restricted) were found to be present in 51% of 264 patients with MM. These protective T cells exhibit telomere-independent senescence, rather than the exhausted or anergic phenotype [176]. Suen et al. demonstrated that PD-1 expression is downregulated in clonal BM cytotoxic T cells, compared with the levels in nonclonal T cells, in MM patients [177]. Thus, the role of PD-1 or PD-L1 blockade needs to be investigated in detail, and clinical trials need to be performed to evaluate its therapeutic potential.

### 5.4. Antibody-Related Therapies

Several antigens that exhibit strong expression in MM cells, including CD38, CD138, CD56, CD74, CD40, insulin-like growth factor-1 receptor (IGF-1R), signaling lymphocyte activating-molecule F7 (SLAMF7), and immunoglobulin superfamily member FcRL5, may be candidates for antibody-related immunotherapy [178]. Numerous naked antibodies have been tested in preclinical myeloma models, and antibodies against six antigens, that is, CD38, CD74, CD40, SLAMF7, IL-6, and IGF-1R, have been examined in clinical trials. Daratumumab [179] and SAR650984 [180] are anti-CD38 monoclonal antibodies that have shown satisfactory response rates in patients with relapse/refractory MM and CD38^+^ hematological malignancies (including 27 patients with MM) in separate phase I clinical trials. A phase II study of daratumumab plus proteasome inhibitor in patients with IMiD refractory myeloma and a phase I/II study of the combination of lenalidomide and dexamethasone are currently underway [181, 182]. SAR650984 is currently being tested in a phase I dose-escalation study and a phase Ib combination study with lenalidomide and dexamethasone [183, 184]. Milatuzumab [185], an anti-CD74 monoclonal antibody, resulted in only 26% of patients achieving stable disease (SD), with a 0% overall response rate (ORR) in patients with refractory/relapse MM. Dacetuzumab [186] and lucatumumab [187] are anti-CD40 monoclonal antibodies that yielded ORRs of 0% (20% of patients achieving SD) and 4% (43% of patients achieving SD), respectively. However, there are no trials currently underway in patients with MM. Elotuzumab, an anti-SLAMF7 (CS1) monoclonal antibody, yielded no objective responses in a phase I clinical trial [188]. However, the combination of elotuzumab, lenalidomide, and dexamethasone yielded an ORR of 84% in patients with refractory/relapse MM in a phase II clinical trial [189]. In a recent phase III study, 321 patients with relapse/refractory MM received elotuzumab plus lenalidomide and dexamethasone, and 325 patients with relapse/refractory MM received the control treatment of lenalidomide and dexamethasone. After a median follow-up of 24.5 months, the rates of progression-free survival (PFS) at 1 and 2 years were 68% and 41%, respectively, in the elotuzumab group as compared with 57% and 27%, respectively, in the control group. Median PFSs were 19.4 and 14.9 months in the elotuzumab and control groups, respectively, and the ORRs were 79% and 66% in the elotuzumab and control groups, respectively [190]. The anti-IGF-1R antibody figitumumab (CP-751871) and AVE 1642 showed disappointing results in phase I studies [191, 192]. However, treatment with the IGF-1R inhibitor OSI-906 or transfection with IGF-1R-targeting small hairpin RNA had synergistic effects on bortezomib sensitivity in cell lines and patient samples [193]. Siltuximab, another monoclonal antibody targeting IL-6, had minimal effects in a phase I study [194, 195] and exhibited no benefits in a phase II clinical trial in patients with refractory/relapse MM [196].

Another type of antibody-related therapy is antibody-drug-conjugated therapy. The anti-CD138 antibody-drug conjugate (ADC), indatuximab ravtansine (BT062), had an ORR of 11%, with 41% achieving SD, in 27 patients with relapse/refractory MM in a phase I study [197]. Combined with lenalidomide and dexamethasone, this ADC resulted in an ORR of 78% in nine patients [198]. The anti-CD56 ADC lorvotuzumab, mertansine, yielded an ORR of 17%, with 28% achieving SD, in selected patients with MM exhibiting CD56 expression in a phase I study [199]. A few additional ADCs are currently being examined in preclinical studies, including ADCs targeting CD74, Fc receptor-like 5 (FcRL5), and B-cell maturation antigen (BCMA). Milatuzumab, an anti-CD74 antibody conjugated to doxorubicin, shows* in vitro* and* in vivo* activity against MC/CAR cells and MC/CAR xenografts in SCID mice [200]. The anti-FcRL5 maytansine analog (DM4) and monomethyl auristatin E (MMAE) have activities similar to those of bortezomib (biweekly treatment) in the inhibition of tumor growth in subcutaneous xenografts of OPM2-FcRL5 and EJM-FcRL5 cells in SCID mice and have been shown to be well tolerated in monkeys in a preclinical study [201]. An anti-BCMA antibody conjugated to monomethyl auristatin F (MMAF) has been reported to show rapid internalization, efficient trafficking to lysosomes, and high antigen recycling rates by 6 h after administration [202]. The anti-BCMA ADC GSK2857916 also resulted in elimination of xenografts arising from myeloma cells [203].

Chimeric antigen receptor- (CAR-) modified T-cell therapy is a new type of immunotherapy. Adoptive transfer of T cells engineered to express chimeric antigen receptors (CARs) can specifically recognize tumor-associated antigens, combining the advantages of non-major histocompatibility complex- (MHC-) restricted recognition with efficient T-cell activation and expansion [204–207]. CARs combine the antigen recognition domain of the antibody with the intracellular domain of the T-cell receptor-*ζ* (TCR-*ζ*) chain or Fc*γ*RI protein into a single chimeric protein that is capable of triggering T-cell activation in a manner very similar to that of the endogenous TCR [208, 209]. CS-1 is a cell surface glycoprotein of the signaling lymphocyte activation molecule (SLAM) receptor family that is highly and selectively expressed on normal plasma cells and MM cells, with lower expression on NK cells and little or no expression on normal tissues. CS1-CAR NK cells exhibit enhanced MM cytolysis and IFN-*γ* production and exhibit tumor suppressive effects on MM cell lines, primary MM tumor cells, and MM xenograft mouse models [210, 211]. CD138 is highly expressed on MM cells and is involved in the development and/or proliferation of these cells [212]. Guo et al. reported that four out of five patients with chemotherapy-refractory MM treated with CART-138 therapy achieved SD longer than 3 months [213]. In a preclinical study, anti-BCMA-CAR-transduced T cells exhibited BCMA-specific functions, including cytokine production, proliferation, cytotoxicity, and* in vivo* tumor eradication. Importantly, anti-BCMA-CAR-transduced T cells recognize and kill primary MM cells [214]. A clinical trial examining CART-19 combined with autologous stem cell transplantation (ASCT) in patients with early refractory/relapse MM is currently underway [215].

### 5.5. Histone Deacetylase Inhibitors [150]

Deacetylases are a group of enzymes that affect various intracellular proteins, including histones, transcription factors, and molecular chaperones, which modulate gene expression, cellular differentiation, and survival [102]. Deacetylase inhibitors (DACi), including panobinostat and vorinostat, have been evaluated for the treatment of MM. The addition of proteasome inhibitors to DACi treatment regimens enhances the sensitivity of MM cells to DACi to induce mitochondrial dysfunction, caspase-9, caspase-8, and caspase-3 activation, and poly (ADP-ribose) polymerase degradation, which is associated with NF-*κ*B inactivation, c-Jun NH_2_-terminal kinase activation, p53 induction, caspase dependent cleavage of p21^CIP1^, p27^KIP1^, and Bcl-2, and cyclin D1 downregulation [216]. The mechanism of this synergistic apoptotic effect on MM cells is multifactorial and includes disruption of protein degradation and inhibition of the interaction of MM cells with the tumor microenvironment [217]. Rocilinostat (ACY-1215) is HDAC6 inhibitor that targets aggressomal protein degradation systems. A synergistic antitumor effect of ACY-1215 and proteasome inhibitors was observed in MM. In addition, a potential benefit was observed in MM-related bone diseases with the combination of these two drugs [218, 219].

### 5.6. Other Agents

Other drugs, including cell signaling targeted therapies (PI3K/AKT/mTOR, p38 MAPK, Hsp90, Wnt, Notch, Hedgehog, and cell cycle) and strategies targeting the tumor microenvironment (hypoxia, angiogenesis, integrins, CD44, CXCR4, and selectins) are candidates for the treatment of refractory and relapse MM [220]. PI3K/AKT is upregulated during refractory and relapse MM. Bortezomib and IMiDs (thalidomide and lenalidomide) do not impact PI3K/AKT signaling [102]. The PI3K/AKT pathway regulates apoptosis, cell cycle, and tumor proliferation [221]. AKT indirectly activates mTOR, a complicated checkpoint of cellular growth influenced by growth factor signaling, adenosine monophosphate levels, and nutrient and O_2_ availability [222]. Perifosine (KRX-0401) is an oral bioactive alkylphospholipid that is thought to target cell membranes and modulate multiple signaling pathways, including the inhibition of AKT and promotion of apoptosis in MM cells [223]. A phase I study with perifosine in combination with lenalidomide and dexamethasone [224] and a phase I/II study with perifosine in combination with bortezomib with or without dexamethasone in refractory and relapse MM [225] demonstrated high treatment tolerance and beneficial effects on survival. Rapamycin and some analogues (temsirolimus or CCI-779 and everolimus or RAD001) are inhibitors of mTOR and have shown preclinical potential as MM therapies. Phase I/II clinical trials using temsirolimus and everolimus in heavily pretreated MM patients showed high tolerance and acceptable response rates [226, 227]. NVP-BEZ235 is a dual pan inhibitor of the PI3K/AKT/mTOR pathways at the levels of PI3K and mTOR, which inhibits growth and proliferation in MM. Moreover, synergism studies have revealed synergistic and additive effects of NVP-BEZ235 in combination with melphalan, doxorubicin, and bortezomib [228]. P38 is constitutively activated in human myeloma and has been implicated in osteoclast and osteoblast activity and bone destruction [229]. The effect of a p38 alpha-selective MAPK inhibitor, SCIO-469 (indole-5-carboxamide, ATP-competitive inhibitor), or its structural analogue, SD-282 (indole-5-carboxamide, ATP-competitive inhibitor), reduced human myeloma cell growth* in vivo* at early and advanced phases of the disease; the same study also provided evidence of the potential for cotherapy with dexamethasone in mouse models of MM [230]. However, LY2228820, a p38 MAPK inhibitor, significantly enhanced toxicity in MM patients [231]. Therefore, more studies on this pathway are required for the development of safe and effective compounds.

Tanespimycin, an Hsp90 inhibitor, reduces tumor cell survival* in vitro* by affecting the IL-6 receptor and elements of the PI3K/AKT and MAPK signaling pathways, through abrogation of the protective effect of BMSCs. Tanespimycin is known to inhibit angiogenesis [232]. A phase I/II study with tanespimycin and bortezomib in relapse/refractory MM patients showed acceptable toxicity and durable response rates [233].

Cancer stem cells use many of the same signaling pathways that are found in normal stem cells, such as Wnt, Notch, and Hedgehog (Hh). Agents targeting these pathways would complement current treatment approaches [234–237]. Other agents targeting the cell cycle, such as seliciclib [238] and LCQ195 [239], cyclin D kinase (CDK) inhibitors, and MLN8237 [240], an aurora-A kinase inhibitor, have demonstrated therapeutic benefits in MM in a preclinical setting.

TNF-related apoptosis-inducing ligand or Apo ligand (TRAIL/Apo2L) is a member of a superfamily of cell death-inducing ligands which also includes TNF-*α* and Fas ligand (FasL or CD95L) [241]. In a preclinical study, TRAIL/Apo2L selectively induced apoptosis in human MM cells, including cells that were sensitive or resistant to dexamethasone and doxorubicin [78], and reversed the bortezomib-induced upregulation of *β*-catenin, MCL1, and FLIP, thereby enhancing the cytotoxicity of combination therapy [242]. This treatment may represent a promising candidate for targeted therapy.

In MM, the impacts of tumor microenvironmental factors such as hypoxia, angiogenesis, and interactions between MM and BMSCs have become an important consideration for understanding disease progression and resistance to therapy and have been incorporated into novel drug screening approaches. VEGFR antagonists inhibit angiogenesis in the MM microenvironment [243]. However, the clinical data for VEGFR antagonists, including pazopanib [244], vandetanib [245], and SU5416 [246], have demonstrated disappointing results.

SDF-1 is produced by BM-derived stromal cells, and its receptor CXCR4 is expressed on the surface of normal and MM cells. The SDF-1/CXCR4 axis is a key regulator of MM cell homing, adhesion, and motility [247]. The CXCR4 antagonist AMD3100 was shown to block MM cell interactions with the BM microenvironment and consequent signaling responses, leading to enhanced sensitivity to therapy [248]. In a phase I trial of plerixafor and bortezomib as a chemosensitization strategy in relapse or relapse/refractory MM patients, preliminary results showed that the combination is well tolerated and demonstrates an acceptable response rate [249].

Mitochondria are important organelles involved in apoptosis under conditions of oxidative stress. Chauhan et al. reported that combining PK-11195, an antagonist of the mitochondrial peripheral benzodiazepine receptors (PBRs), with bortezomib triggers synergistic anti-MM activity, even in MM cells resistant to doxorubicin, melphalan, thalidomide, dexamethasone, and bortezomib. The mechanism through which apoptosis is induced includes loss of mitochondrial membrane potential, superoxide generation, release of the mitochondrial proteins cytochrome-c and Smac, activation of caspase-8/caspase-9/caspase-3, and activation of c-Jun NH_2_-terminal kinase (JNK) [250].

## 6. Conclusion

Based on a thorough understanding of the mechanism and importance of the MM microenvironment, proteasome inhibitors, such as bortezomib, have been developed in combination with IMiDs and steroids to provide dramatic improvement in treatment response and survival in MM patients. However, MM is still an incurable disease. The possible mechanisms of drug resistance include* MDR* gene polymorphism and p-glycoprotein overexpression in MM cells, microenvironmental changes (cell adhesion, activation of cytokine-related antiapoptosis pathways such as the JAK/STAT and PI3K/AKT pathways), clonal evolution such as hyperexpression of the proteasome-related gene,* PSMD4*, related to chromosome 1q21 amplification, t(4;14) unbalanced translocation, and selected CD34^+^CD138^+^B7-H1^+^CD19^−^ plasma cell accumulation after treatment. The up- and downregulation of various miRNAs modulate MM cell survival, cell cycle, and microenvironment, thereby contributing to drug resistance, including against bortezomib. PD-1 is enriched on T cells in MM patients, and PD-L1 expression on MM cells is enhanced. PD-1/PD-L1 interactions have been shown to mediate tumor escape from immune control in a number of animal models. Moreover, PD-1/PD-L1 interactions are related to immune dysfunction in MM patients. PD-L1 in MM cells and PD-1 in T cells surrounding tumors contribute to drug resistance mechanisms. Potential therapies, including second-generation proteasome inhibitors, new immunomodulatory agents, DACi, and kinase inhibitors such as the mTOR inhibitor, as well as drugs targeting cytokine-related pathways, anti-PD-1/anti-PD-L1 monoclonal antibodies, and monoclonal antibodies (naked or conjugated with drugs), and CAR-T therapy, are under preclinical and clinical investigation to provide better treatment responses in MM patients. Study of the pathophysiology of MM and the mechanisms of drug resistance will enable the development of novel therapeutic strategies to cure this disease. Further clinical trials of the novel agents described here are also necessary, especially for refractory/relapse MM patients.
